# Differential Expression and Co-Variation of HERV-Associated Transcripts, IL-17 Cytokines, and NFκB in Paired Colorectal Cancer Tissues

**DOI:** 10.3390/biomedicines14081741

**Published:** 2026-08-01

**Authors:** Vlad-Alexandru Ionescu, Gina Gheorghe, Claudiu Stefan Turculet, Teodor Florin Georgescu, Razvan Matei Bratu, Ioana-Alexandra Baban, Cristina Mambet, Carmen Cristina Diaconu, Valentin Enache, Laura Denisa Dragu, Ana Iulia Neagu, Ioana Madalina Pitica, Marian Constantin, Camelia Cristina Diaconu, Coralia Bleotu

**Affiliations:** 1 Faculty of Medicine, University of Medicine and Pharmacy Carol Davila Bucharest, 050474 Bucharest, Romania; vladalexandru.ionescu92@gmail.com (V.-A.I.);; 2 Internal Medicine Department, Clinical Emergency Hospital of Bucharest, 105402 Bucharest, Romania; 3 General Surgery Department, Clinical Emergency Hospital of Bucharest, 105402 Bucharest, Romania; 4 Department of Cellular and Molecular Pathology, Stefan S. Nicolau Institute of Virology, Romanian Academy, 030304 Bucharest, Romania; 5 Hematology Department, Emergency University Clinical Hospital of Bucharest, 050098 Bucharest, Romania; 6 Department of Anatomical Pathology, Clinical Emergency Hospital of Bucharest, 105402 Bucharest, Romania; 7 Research Institute of the University of Bucharest (ICUB), University of Bucharest, 060023 Bucharest, Romania; 8 Department of Microbiology, Institute of Biology of the Romanian Academy, 060031 Bucharest, Romania; 9 Academy of Romanian Scientists, 050085 Bucharest, Romania

**Keywords:** colorectal cancer, HERV, ERVWE1, IL-17E, NFκB

## Abstract

**Background**: The mechanistic link between HERV-associated transcript expression and the inflammatory pathways involved in colorectal cancer (CRC), particularly the IL-17 axis, remains insufficiently characterized. We performed an exploratory paired-tissue study to investigate whether HERV-associated transcript deregulation in CRC aligns with a distinct local inflammatory signature, potentially consistent with endogenous retroviral re-expression linked to local immune activation and viral mimicry-related processes within the tumor microenvironment. **Methods**: Matched colorectal tumor tissue (T) and histologically tumor-free adjacent tissue (N) were obtained from 21 treatment-naive CRC patients. Expression of selected HERV-associated loci/transcripts (HERV-FRD, HERV-R, HERV-K, HERV-V1, HERV-1, HERV-H, and ERVWE1), HHLA1/HHLA2, IL-17 family members, and NFKB1 was assessed by RT–qPCR. **Results**: CRC tumors exhibited selective upregulation of HERV-FRD, HERV-R, HERV-K, HERV-V1, ERVWE1, HHLA1, and HHLA2, whereas HERV-1 and HERV-H showed no significant paired differences. IL-17E was also increased in tumor tissue, while NFKB1 did not show significant paired variation. Paired differential expression analysis [Δ(T−N)] identified a coordinated HERV/ERVWE1 signature that positively correlated with IL-17E after FDR correction, whereas HHLA1/HHLA2 displayed partially distinct behavior. No consistent associations were observed with clinicopathological variables. **Conclusions**: CRC appears to harbor a selective HERV/ERVWE1-associated transcriptional program linked to IL-17E, supporting a candidate inflammatory axis that may reflect a biologically relevant component of the tumor microenvironment. This expression pattern may also be compatible with a localized endogenous retroviral reactivation state linked to viral mimicry-related inflammatory signaling, although this interpretation remains indirect and requires mechanistic validation. These findings are associative and hypothesis-generating, but they provide a conceptual basis for future mechanistic validation and for exploring whether HERV-linked inflammatory signatures may have biomarker or therapeutic relevance in CRC.

## 1. Introduction

Colorectal cancer (CRC) is currently the third most frequently diagnosed malignancy in adults and the second leading cause of cancer-related mortality worldwide [1]. Despite substantial advances in early detection and in diagnostic and therapeutic management, the 5-year overall survival of CRC patients remains approximately 65%, with marked differences between screen-detected cancers and those diagnosed outside screening programs (83.4% vs. 57.5%) [2]. In light of these data, recent studies have increasingly focused on CRC etiology, with particular attention to the intestinal microbiome and human endogenous retroviral elements (HERVs) [3,4]. However, whether HERV-associated transcript reactivation in CRC is linked to a distinct inflammatory program within the tumor microenvironment, particularly involving the IL-17 axis, remains insufficiently defined [3,4]. Accordingly, the present study was designed to explore whether selected HERV-associated transcripts exhibit coordinated deregulation in colorectal tumor tissue relative to matched tumor-free adjacent tissue, and whether this pattern aligns with IL-17 family cytokines and NFκB-related signaling.

HERVs constitute approximately 8% of the human genome [5]. Their contribution to oncogenesis remains controversial and has been attributed either to epigenetic dysregulation or to promoter/enhancer activity influencing oncogene expression [6]. Mechanistically, HERVs may affect gene regulation and protein output through multiple routes, including: (i) direct production of proteins encoded by internal HERV sequences (Env, Gag, Pol); (ii) onco-exaptation events involving functional interactions between HERV sequences and host genes; and (iii) HERV-derived long non-coding RNAs (lncRNAs) capable of modulating gene expression [6,7,8]. These mechanisms are of potential relevance in CRC, where epigenetic deregulation, inflammatory signaling, and tumor–microenvironment interactions are recognized contributors to disease progression. 

In addition, HERVs may contribute to tumorigenesis by shaping the tumor microenvironment (TME) and its immune contexture, which are key determinants of patient prognosis [9]. HERV-derived peptides have been proposed as a potential source of tumor-associated antigens, and HERV re-expression in tumor cells can elicit a “viral mimicry” state [10,11,12]. Viral mimicry refers to an antiviral-like cellular program induced by endogenous stimuli, characterized by reactivation of epigenetically silenced HERVs and induction of interferon signaling, thereby modulating antitumor immunity [11]. Within this response, TANK-binding kinase 1 (TBK1) promotes dimerization and nuclear translocation of interferon regulatory factor 7 (IRF7) via phosphorylation, forming an active transcriptional complex that initiates type I and type III interferon responses and amplifies antiviral and immunomodulatory host functions [13]. A higher global HERV burden has been associated with increased immune infiltration across multiple cancer types, including CRC [14,15]. The immunological relevance of HERV expression is further supported by the reported association between HERV levels and improved survival among patients with urothelial carcinoma treated with immune checkpoint inhibitors [16]. Moreover, several HERV-associated transcripts, including HERV-K, HERV-R, HERV-FRD, and ERVWE1, have been implicated in cancer-related transcriptional deregulation, tumor cell plasticity, and immune modulation, supporting their investigation in CRC as candidate components of a biologically relevant tumor-associated expression program.

Particularly in the context of autoimmune diseases, direct mechanistic relationships between HERV reactivation and polarization of the immune response, including through IL-17-producing pathways, have been demonstrated. For example, in autoimmune hepatitis, the HERV1-env protein (from the viral envelope) induces endoplasmic reticulum stress and activates the ATF6 pathway of the UPR response. Activated ATF6α binds directly to the promoters of the RORC and STAT3 genes, increasing their expression and leading to the production of IL-17A. Inhibition of HERV1-env restores regulatory T cell function [17]. In systemic lupus erythematosus, increased expression of HERV-E clone 4-1 in CD4+ T cells correlates with disease activity. The 3′LTR region of HERV-E acts as a miR-302d-sniffing RNA. This leads to the derepression of MBD2, a protein involved in DNA hypomethylation, favoring the release of IL-17 [18]. On the other hand, in severe SARS-CoV-2 infection, a simultaneous increase in the expression of HERV-K, HERV-W, and IL-17 (along with IL-6, TNF-α) in the nasal mucosa was observed. HERVs contribute to the amplification of the inflammatory response, including IL-17-mediated pathways [19]. Although these observations derive from disease contexts distinct from CRC, they support the biological plausibility of a link between endogenous retroviral reactivation and IL-17-associated inflammatory signaling. This issue is particularly relevant in CRC, where mucosal immunity, microbial interactions, and inflammatory circuits are closely involved in tumor development and progression.

Against this background, the aim of the present study was to characterize the expression profile of selected HERV-associated transcripts in colorectal tumor tissue compared with matched tumor-free adjacent tissue and to explore their relationships with the IL-17 cytokine family and NFκB-related signaling, given their proposed roles in tumor-associated inflammation and CRC progression. In this exploratory paired-tissue study, we profiled a focused panel of HERV-associated transcripts/loci selected to capture candidate markers previously implicated in endogenous retroviral re-expression, immune modulation, or cancer-related transcriptional deregulation, including HERV-FRD, HERV-R, HERV-K, HERV-V1, HERV-1, HERV-H, and endogenous retrovirus group W member 1 envelope (ERVWE1), together with the HERV-associated immune checkpoint–related transcripts HERV-H long terminal repeat-associating protein 1 (HHLA1) and HERV-H long terminal repeat-associating protein 2 (HHLA2). In parallel, we quantified IL-17 family members (IL-17A–F) and NFκB to capture complementary inflammatory pathways potentially linked to endogenous retroviral re-expression. By using a paired tumor/tumor-free adjacent tissue design, we sought to reduce inter-individual background variability and better isolate tumor-associated expression shifts. Through this integrated profiling strategy, the study aims to contribute to clarifying the potential biological relevance of HERV re-expression in CRC and to generate hypotheses regarding its interplay with IL-17– and NFκB-associated inflammatory signaling. More specifically, we aimed to determine whether CRC harbors a selective HERV-associated transcriptional signature linked to a distinct local inflammatory milieu rather than a nonspecific global deregulation pattern. Accordingly, all findings should be interpreted within an exploratory and hypothesis-generating framework.

## 2. Materials and Methods

### 2.1. Study Design and Population

We conducted an exploratory, single-center study including 21 patients with colorectal cancer (CRC) who underwent surgical resection with curative or palliative intent and had not received prior oncologic treatment. The study was carried out over a 3-year period (February 2023–October 2025) at the Bucharest Emergency Clinical Hospital and the “Ștefan S. Nicolau” Institute of Virology, Bucharest, within the framework of a research collaboration agreement.

All participants provided written informed consent for the use of clinical data and biological samples for research purposes. The study was conducted in accordance with the Declaration of Helsinki and was approved by the Ethics Committee of the Bucharest Emergency Clinical Hospital (approval no. 1400, dated 7 February 2023).

**Figure 1:**
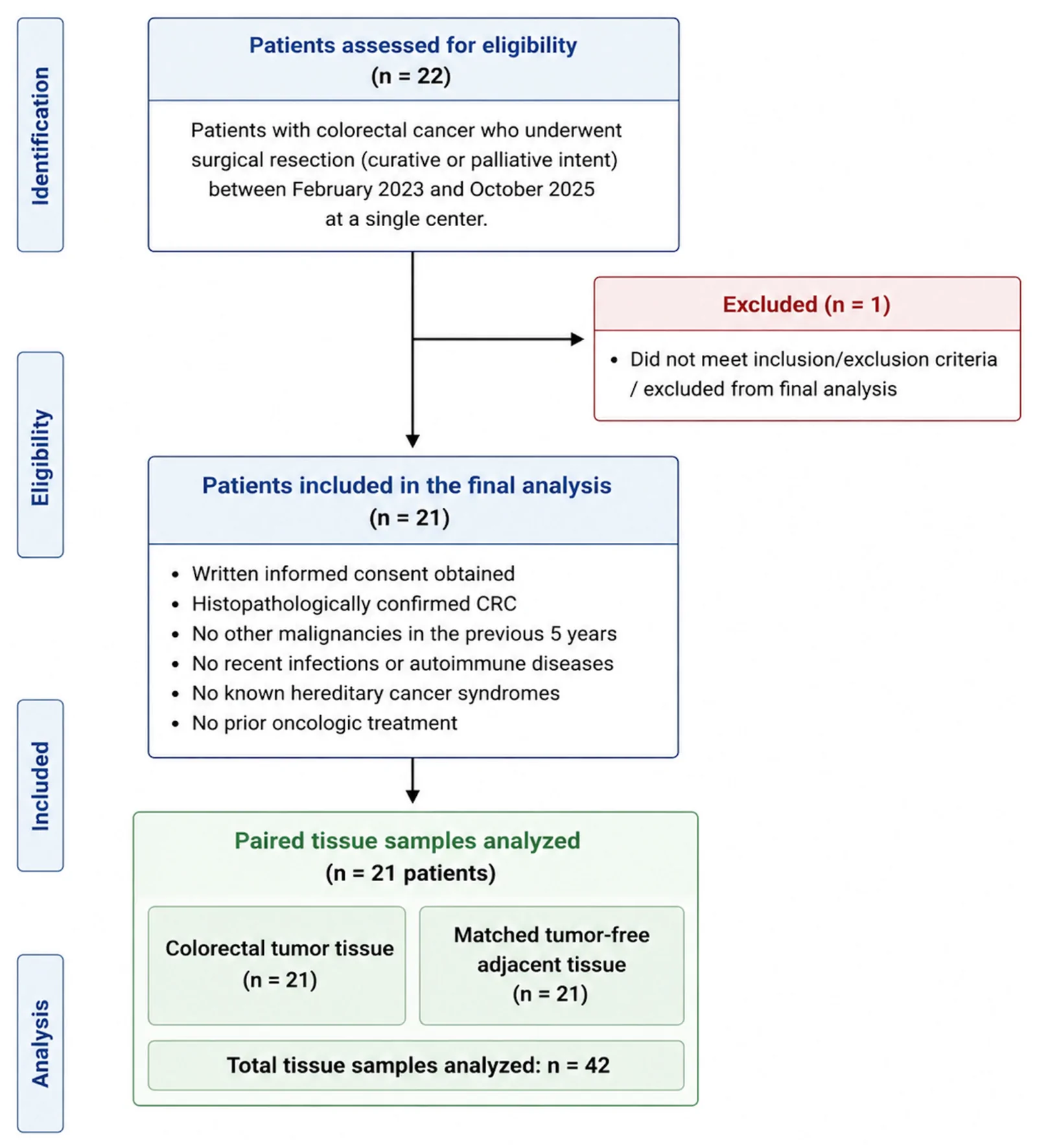
Flow diagram of patient selection and inclusion in the final analysis.

Inclusion criteria were: signed informed consent, histopathologically confirmed CRC, absence of other malignancies at a different site within the previous 5 years, and absence of recent infections or autoimmune diseases (Figure 1). Exclusion criteria were: refusal to sign informed consent, concomitant malignant or autoimmune diseases, and recent infections (Figure 1). One patient initially enrolled was subsequently excluded from the final analysis. None of the included patients had known hereditary cancer syndromes, and none had been previously exposed to oncologic therapies (Figure 1). Demographic, clinical, and paraclinical data were recorded in an electronic database (Microsoft Excel) and subsequently analyzed statistically.

### 2.2. Sample Collection

For each included patient, paired samples of tumor tissue and matched tumor-free adjacent tissue were collected from the surgical resection specimen. Adjacent samples were taken ≥5 cm from the macroscopic tumor margin and were confirmed as tumor-free on routine histopathology.

Immediately after surgical excision, the specimens were transported to the pathology laboratory, where a pathologist obtained the tissue samples using sterile Eppendorf tubes without preservation medium, prior to fixation of the surgical specimen in formalin.

Samples were transported to the “Ștefan S. Nicolau” Institute of Virology, Bucharest, within a maximum of 2 h after collection and stored at −80 °C until completion of study recruitment.

Histopathological evaluation was performed on the surgical resection specimens by an experienced pathologist. Tumors were classified according to the World Health Organization (WHO) Classification of Tumours of the Digestive System, 5th edition. Histological type and tumor grade were recorded, and pathological staging was established according to the TNM classification. The cohort included conventional-type adenocarcinomas and adenocarcinomas with a mucinous component, classified as well, moderately, or poorly differentiated tumors (G1–G3). Matched adjacent tissue samples were confirmed histologically to be tumor-free.

### 2.3. Total RNA Extraction

Total RNA was extracted from tumor fragments using TRIzol™ reagent (Invitrogen, Thermo Fisher Scientific, Waltham, MA, USA), according to the manufacturer’s protocol. Briefly, tumor fragments were minced and homogenized in TRIzol reagent for cell lysis and RNase inactivation. After incubation, complete dissociation of nucleoprotein complexes was achieved by phase separation with chloroform, followed by centrifugation. The aqueous phase containing RNA was transferred to a new tube, and RNA was precipitated with isopropanol. The RNA pellet was washed with 75% ethanol, air-dried, and resuspended in RNase-free water. To minimize the risk of genomic DNA contamination, the RNA samples were treated with DNase (Turbo DNase, Thermo Fisher Scientific, according to the manufacturer’s instructions) prior to downstream reverse transcription analysis. RNA concentration and purity were assessed spectrophotometrically (A260/A280 ratio), and integrity was verified by agarose gel electrophoresis to ensure the presence of intact ribosomal bands (28S and 18S). Samples showing inadequate purity ratios were re-extracted until optimal RNA quality parameters were achieved. 

### 2.4. Reverse Transcription (cDNA) Using High-Capacity cDNA Reverse Transcription Kit

The cDNA synthesis (cDNA) was performed using the High-Capacity cDNA Reverse Transcription Kit (Applied Biosystems), according to the manufacturer’s instructions. Thus, 2 µg of total RNA, reaction buffer, dNTPs, random primers, RNase inhibitor and reverse transcriptase were subjected to the reverse transcription reaction performed in a thermal cycler, using the following thermal program: 25 °C for 10 min for priming, 37 °C for 120 min for cDNA synthesis, followed by 85 °C for 5 min for enzyme inactivation, with final holding at 4 °C. The obtained cDNA was subsequently diluted and used as a template for real-time PCR reactions or stored at −20 °C until use.

### 2.5. Real-Time PCR Using Maxima SYBR Green qPCR Master Mix

Target gene expression was analyzed by real-time PCR using Maxima SYBR Green qPCR Master Mix (Thermo Fisher Scientific, Waltham, MA, USA), according to the manufacturer’s protocol. The reactions contained: Master Mix 2× (with DNA polymerase, dNTPs, SYBR Green and MgCl_2_), target gene-specific primers, cDNA as template, nuclease-free water. The following primers pre-validated by Origene Technologies were used: HERV-FRD/ERVFRD-1 (KU HP219802); ERVWE1/ERVW-1 (SKU HP210993); HERV-H/HHLA1/2 (SKU HP227654); HERV-K/ERVK-6 (SKU HP201793); HERV-R/ERV3-1 (SKU HP201802); HERV-V1/ERVV-1 (SKU HP217732); HERV1 (ALR/GFER) (SKU HP208432); IL17A (SKU HP205926); IL17B (SKU HP210891); IL17C (SKU HP210445); IL17D (SKU HP216891); IL17E/IL25 (SKU HP214569); IL17F (SKU HP216499); NFKB1/NFκB (SKU HP207409) (Table 1). Amplification was performed in a compatible real-time PCR detection system, using the following thermal program: initial activation of the polymerase at 95 °C for 10 min, followed by 40 cycles consisting of denaturation at 95 °C for 15 s, annealing and extension at 60 °C for 60 s. At the end of each qPCR run, melting-curve analysis was performed by gradually increasing the temperature from 60 °C to 95 °C and was used as the primary assay-level specificity control. Agarose gel verification of the final qPCR amplicons for the analyzed targets was performed for randomly selected samples; therefore, amplification specificity was assessed mainly based on melting-curve profiles and the use of commercially pre-validated primer pairs. Each sample was analyzed in two technical replicates. Relative gene expression was calculated using the comparative Ct method (2^−ΔΔCt^), normalized to a constitutive reference gene GAPDH. For statistical analyses and plotting, expression values were reported on a log2 scale as log2(2^−ΔΔCt^) = −ΔΔCt (i.e., log2 relative quantity, log2RQ). Accordingly, within-patient paired differential expression was defined as Δ(T−N) = [−ΔΔCt]_T − [−ΔΔCt]_N, and fold change (FC) was derived as 2^{Δ(T−N)} [20]. GAPDH was selected as the reference gene based on its widespread use in RT-qPCR studies involving colorectal tissue [21]. In the present exploratory study, GAPDH was the only reference gene used for normalization. In our dataset, GAPDH Ct values showed limited variation across samples, with a mean Ct value of 20.5 ± 0.65 (SD), supporting its suitability for internal normalization.

### 2.6. Statistical Analysis

Statistical analyses were performed in R version 4.4.2 (R Foundation for Statistical Computing, Vienna, Austria). All inferential analyses were conducted using paired samples of colorectal tumor tissue (T) and matched tumor-free adjacent tissue (N) for each patient.

Gene expression levels were summarized descriptively as medians with interquartile ranges (IQR). Paired differential expression was calculated as Δ(T−N). Fold change (FC) was derived as 2^{Δ(T−N)} and reported as median (IQR) where applicable.

Given the non-Gaussian distribution of expression data and the relatively small sample size, comparisons between tumor and matched tumor-free adjacent tissue were performed using non-parametric paired tests. A value was classified as non-detectable (ND) when no specific amplification signal was obtained under the predefined qPCR run conditions (including assessment of amplification and melting curve profile). ND values were treated as missing for inferential analyses and were not imputed. For paired expression comparisons using the Wilcoxon signed-rank test, only eligible pairs with detectable values in both tumor (T) and matched normal (N) tissues were included. For markers with differential detectability across tissues, paired detectability (detected vs. ND) was analyzed using the exact McNemar test.

To investigate relationships between biomarkers, correlations were calculated using Spearman’s rank correlation coefficient (ρ) on paired differential expression values Δ(T−N), an approach intended to mitigate inter-individual baseline variability and to capture the tumor-associated component of expression change. Δ(T−N) values were calculated only for markers/pairs with detectable values in both tissues, and only these eligible Δ values were included in Spearman correlation analyses. Markers with insufficient numbers of eligible paired observations were excluded from the primary inferential correlation analyses. For interpretation purposes, absolute correlation coefficients were classified as weak for |ρ| < 0.30, moderate for |ρ| = 0.30–0.49, and strong for |ρ| ≥ 0.50. Given the relatively small sample size of this exploratory study, moderate correlations were not considered biologically irrelevant, but rather interpreted cautiously as potentially informative associations that require confirmation in larger independent cohorts.

To control for type I error due to multiple testing, *p*-values were adjusted using the Benjamini–Hochberg procedure (false discovery rate, FDR). Results were considered statistically significant at *p*(FDR) < 0.05.

Associations between HERV expression and clinicopathological variables (tumor site, histological type, tumor stage I–IV, and tumor grade G1–G3) were assessed using non-parametric approaches and reported together with effect size measures. For variables with more than two categories (e.g., tumor site), effect size was expressed as epsilon squared (ε^2^). For dichotomous variables, effect size was quantified using the rank-biserial correlation (|r|). For ordinal variables (stage and grade), associations were evaluated using Spearman’s |ρ|. Effect size magnitudes were visualized using a heatmap representation.

All statistical tests were two-sided. The significance threshold was set at *p* < 0.05, and at *p*(FDR) < 0.05 for analyses subject to multiple-testing correction.

## 3. Results

The study cohort included 21 patients with CRC, with a relatively balanced sex distribution. The mean age was 64.2 years, spanning a wide age range (Table 2). Regarding tumor location, lesions were more frequently located in the left colon, whereas right-sided colon and rectal tumors were observed at comparable proportions (Table 2). Histopathological assessment showed a predominance of conventional-type adenocarcinoma (NOS), with most tumors classified as moderately differentiated (G2) (Table 2). With respect to staging, stage II was the most frequent category in the cohort (Table 2). Among comorbidities, hypertension was the most prevalent condition, followed by dyslipidemia and type 2 diabetes mellitus (Table 2).

### Paired Expression Profiling of HERV-Associated Transcripts in Tumor Versus Tumor-Free Adjacent Tissue

All paired tumor tissue–tumor-free adjacent tissue samples were included in the gene expression analyses. Molecular profiling revealed substantial interindividual variability in expression levels, detectable in both tumor tissue and matched tumor-free adjacent tissue. 

**Figure 2:**
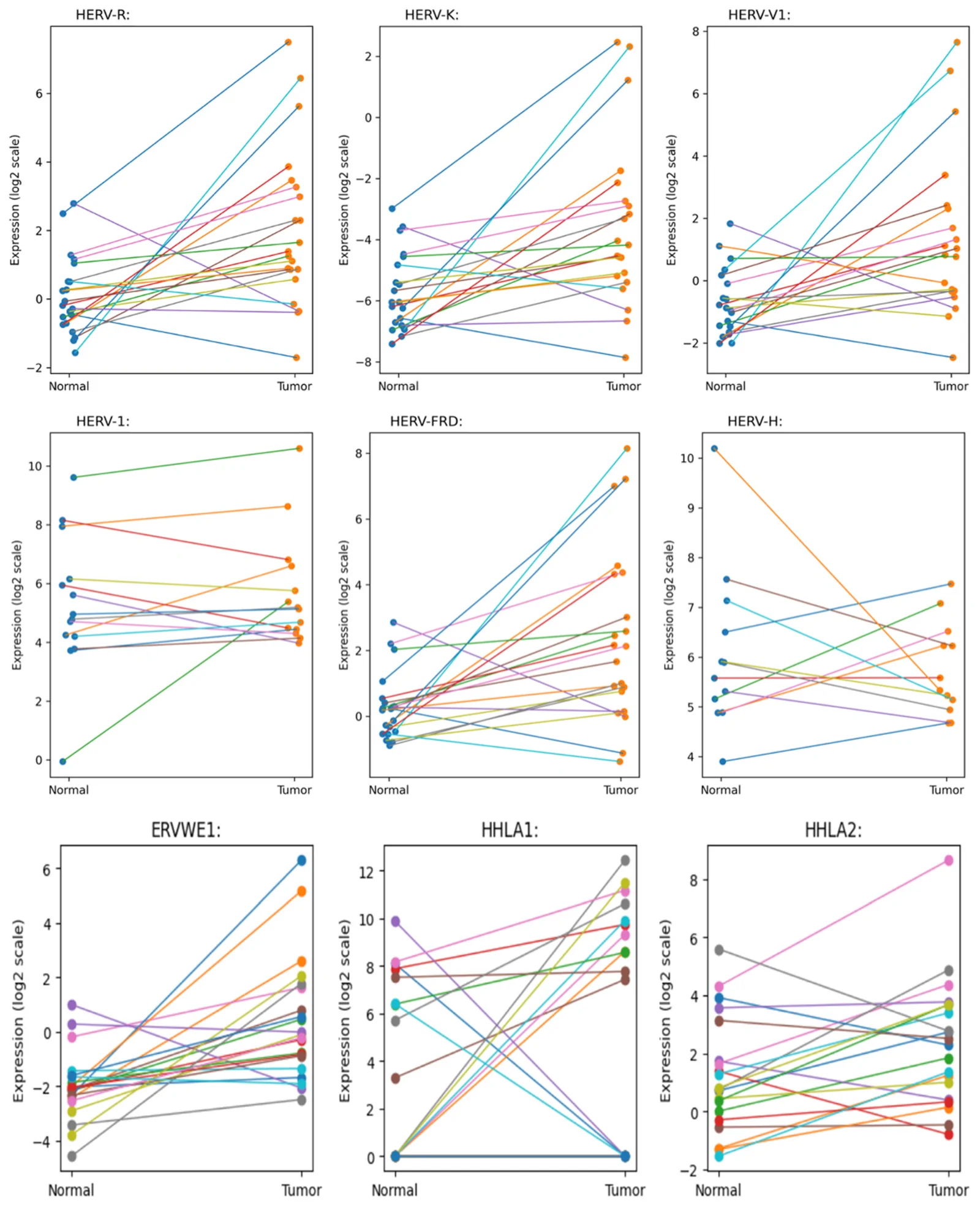
Paired gene expression profiles of HERV-associated transcripts in colorectal tumor (Tumor) and matched tumor-free adjacent tissue (Normal). Each line represents an individual patient. Expression values are shown on a log2 scale.

Paired analyses revealed distinct expression patterns between colorectal tumor tissue and matched tumor-free adjacent tissue for the investigated HERV-associated transcripts (Figure 2). HERV-FRD showed increased expression in tumor tissue and was detectable in all analyzed samples. The paired median difference was positive, indicating higher tumor expression, corresponding to a median fold change (FC) of 3.189; this difference was supported by the Wilcoxon signed-rank test (*p* = 0.002) (Figure 2).

A similar pattern was observed for HERV-R, with higher expression in tumor tissue, reaching statistical significance (Wilcoxon *p* = 0.001) (Figure 2). HERV-K also exhibited a positive paired median difference, and HERV-V1 showed a comparable increase; both differences were statistically significant (Wilcoxon *p* = 0.001 for HERV-K and *p* = 0.003 for HERV-V1) (Figure 2).

Additional paired analyses of ERVWE1, HHLA1, and HHLA2 also showed distinct tumor–normal expression patterns (Figure 2). ERVWE1 showed higher expression in tumor tissue compared with matched tumor-free adjacent tissue, with a positive median paired difference, reaching statistical significance by the Wilcoxon signed-rank test (*p* < 0.001) (Figure 2). HHLA1 also showed higher tumor expression overall, with a significant paired difference (Wilcoxon *p* = 0.031) (Figure 2). Similarly, HHLA2 exhibited increased expression in tumor tissue, with a positive median paired difference, reaching statistical significance (Wilcoxon *p* = 0.027) (Figure 2).

In contrast, HERV-1 and HERV-H did not display consistent differences between tissue types. Although HERV-1 showed a small positive median paired difference, it did not reach statistical significance (Wilcoxon *p* = 0.502) (Figure 2). Likewise, HERV-H demonstrated marked interindividual variability without significant tumor–normal differences (Wilcoxon *p* = 0.970) (Figure 2).

**Figure 3:**
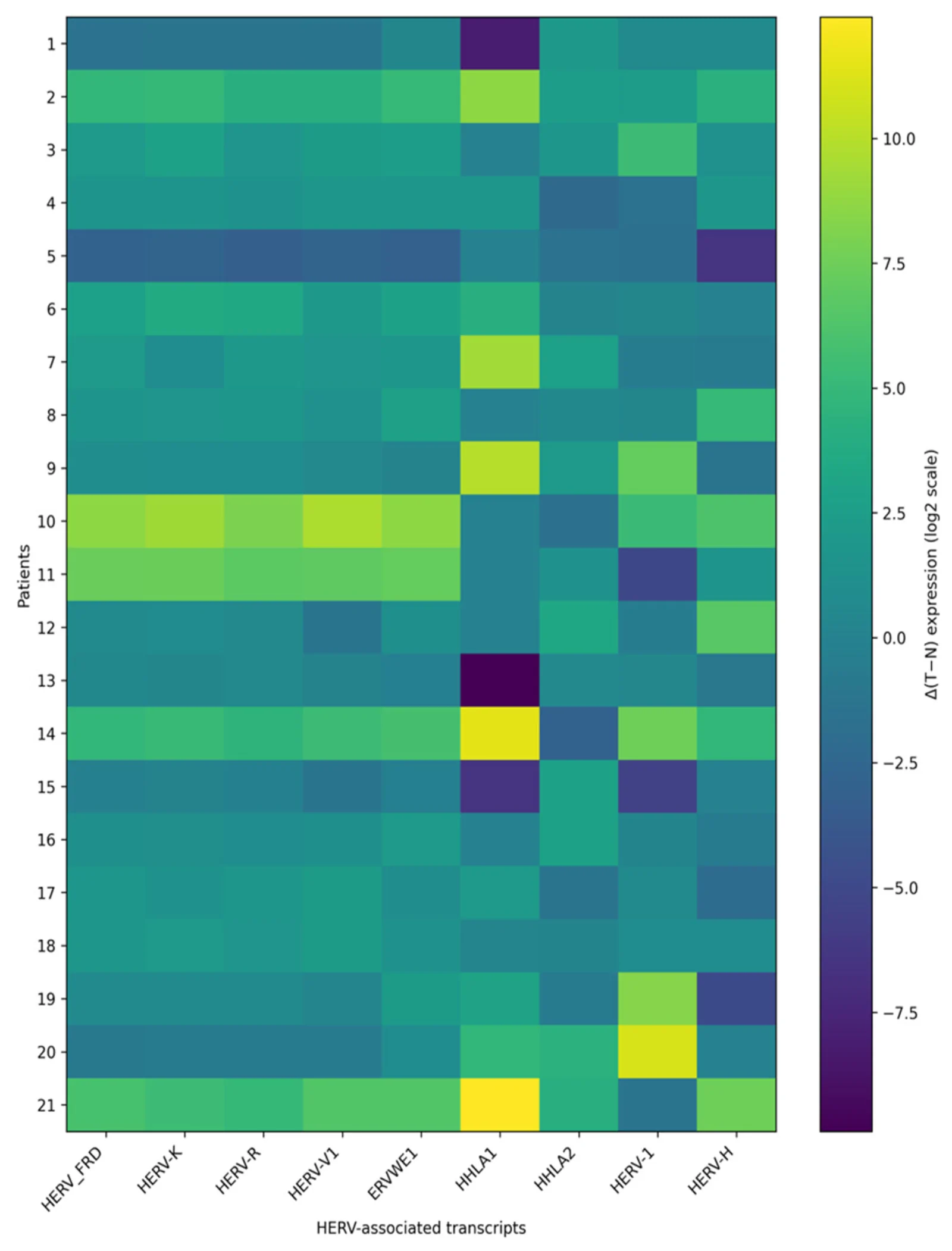
Heatmap of paired differential expression Δ(T−N) for HERV-associated transcripts (log2 scale). Each row represents an individual patient. Color intensity reflects the magnitude of differential expression between tumor (T) and matched tumor-free adjacent tissue (N). Positive values (yellow–green) indicate higher expression in tumor tissue, whereas negative values (blue–purple) indicate lower tumor expression.

To facilitate the visual interpretation of expression changes, a heatmap of the paired differential expression values Δ(T−N) was generated (Figure 3) for the evaluated HERV-associated transcripts (HERV-FRD, HERV-K, HERV-R, HERV-V1, ERVWE1, HHLA1, HHLA2, HERV-1, and HERV-H). Figure 3 highlights considerable interindividual variability in expression profiles, with Δ(T−N) magnitudes varying across patients and transcripts, and more heterogeneous patterns observed for HHLA1/HHLA2 and HERV-1/HERV-H. Overall, HERV-FRD, HERV-K, HERV-R, HERV-V1, and ERVWE1 displayed a predominance of positive Δ(T−N) values, suggesting a general trend toward increased expression in tumor tissue, although the magnitude of differences varied between patients. For HHLA1 and HHLA2, the pattern was more heterogeneous, with variable tumor–normal differences and extreme values in selected cases. Likewise, HERV-1 and HERV-H exhibited more heterogeneous profiles; however, at a descriptive level, Δ(T−N) values were frequently positive, suggesting a potential tendency toward higher tumor expression, although this finding was not statistically significant in the preceding paired analysis.

**Figure 4:**
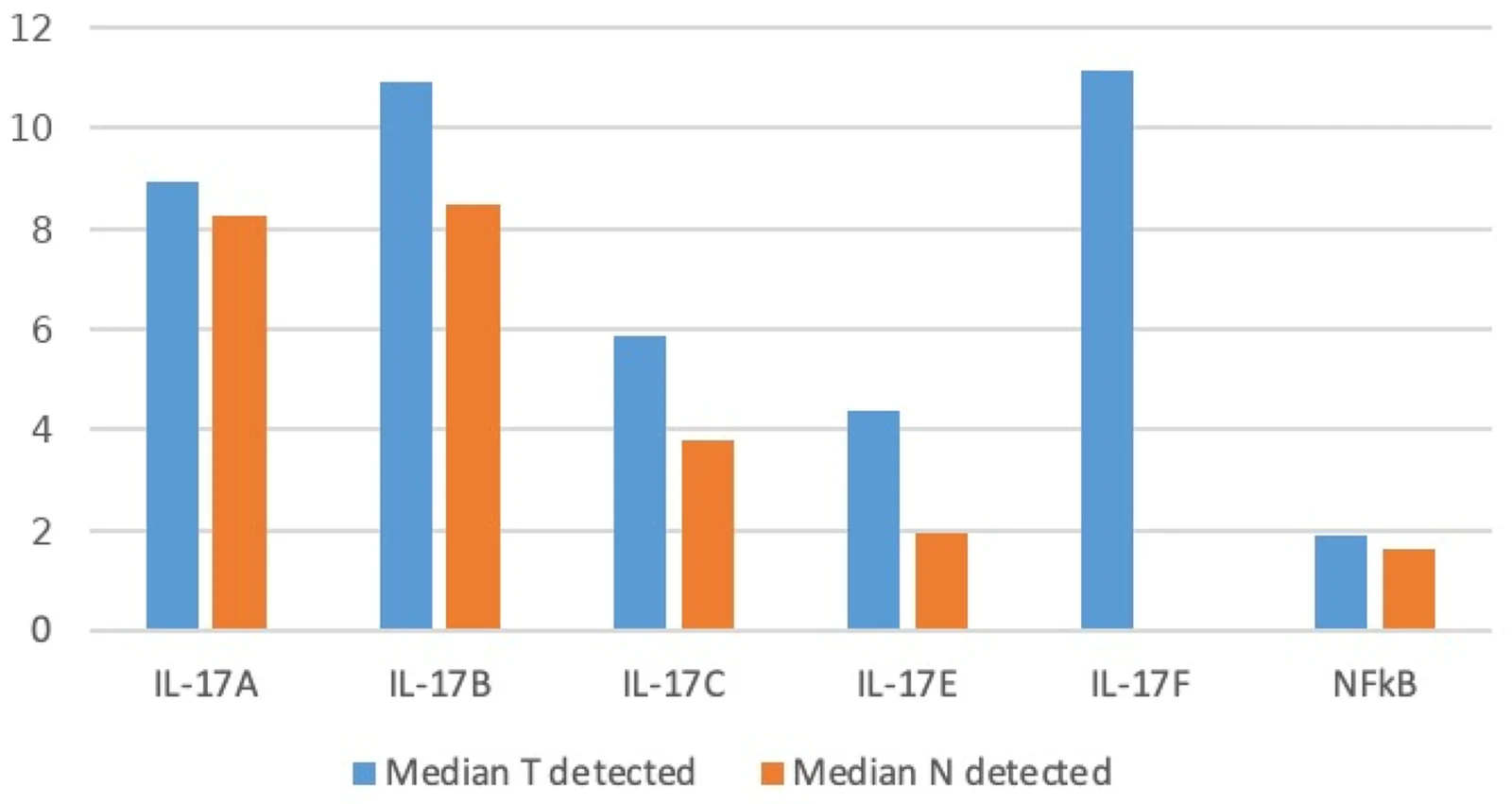
Comparison of median expression between colorectal tumor tissue (T) and matched tumor-free adjacent tissue (N) for IL-17 family members and NFκB. Bars represent median expression among quantifiable values (≥LOD) in tumor and tumor-free adjacent tissue. ND values were excluded from the calculation of medians. Statistical significance was assessed using paired-sample tests and detectability-based analyses (see Table 3).

Subsequently, we performed a comparative analysis of gene expression between colorectal tumor tissue and matched tumor-free adjacent tissue for members of the IL-17 cytokine family and for NFκB (Table 3, Figure 4).

Among the investigated cytokines, IL-17E demonstrated increased expression in tumor tissue, with a positive paired median difference corresponding to a median fold change (FC) of 2.147 (Table 3). This difference was supported by the Wilcoxon signed-rank test for paired samples (*p* = 0.001) (Table 3). IL-17C showed a positive paired median difference, but this trend did not reach statistical significance (Wilcoxon *p* = 0.352) (Table 3).

For IL-17A, IL-17B, and IL-17F, the low number of quantifiable paired observations limited inferential testing; therefore, no Wilcoxon *p*-values were estimated (Table 3). Nevertheless, detectability differed between tissues for IL-17A, with a significant McNemar exact test (*p* = 0.031), indicating higher detectability in tumor tissue than in matched normal tissue. IL-17B and IL-17F also showed a descriptively higher frequency of detection in tumor tissue; however, these differences did not reach statistical significance (McNemar *p* = 1.000 and *p* = 0.250, respectively) (Table 3).

Regarding IL-17A, all patients in whom this cytokine was detected in tumor tissue had conventional-type adenocarcinoma (NOS), predominantly with moderate differentiation (G2) and rectosigmoid localization. Tumor stage among these cases ranged from stage I to stage IV, without a consistent distribution pattern. In contrast, IL-17B expression was identified in tumors arising both in the rectosigmoid region and in the right colon. IL-17F expression was detected exclusively in colorectal tumor tissue samples.

NFκB showed a small positive paired median difference (median Δ(T−N) = 0.253; median FC = 1.192), but this did not reach statistical significance (Wilcoxon *p* = 0.744) (Table 3).

Spearman correlation analysis based on paired differential expression values (ΔT−N) revealed a co-variation pattern across several HERV-associated transcripts, suggesting coordinated deregulation within the tumor context (Table 4). The strongest correlations were observed for HERV-FRD–HERV-K, HERV-FRD–HERV-V1, and HERV-FRD–HERV-R (Table 4). In addition, ERVWE1 showed positive associations with the main tumor-upregulated HERV-associated transcripts, suggesting that ERVWE1 expression shifts may align with the coordinated HERV deregulation observed in this cohort (Table 4). In contrast, correlations involving HERV-1, HERV-H, HHLA1, and HHLA2 were generally small-to-moderate and did not remain significant after FDR correction (Table 4).

Regarding inflammatory markers, IL-17E displayed positive correlations with several HERV-associated transcripts (HERV-FRD, HERV-V1, HERV-K, HERV-R and ERVWE1; all *p*(FDR) = 0.006). By comparison, correlations involving IL-17C were of lower magnitude and did not remain significant after FDR correction across the full testing block (Table 4). Correlations involving IL-17A, IL-17B, IL-17D, and IL-17F were not included in the main inferential analysis due to limited detectability and/or an insufficient number of eligible paired Δ(T−N) values.

NFκB Δ(T−N) showed weak and inconsistent correlations with HERV-associated differential expression signals, and none remained significant after FDR correction. Specifically, NFκB showed weak positive trends with HERV-FRD and HERV-R, and small associations with IL-17E and IL-17C (Table 4). Overall, these data suggest that, within this exploratory cohort, NFκB Δ(T−N) did not co-vary robustly with the main HERV transcripts identified by the strongest correlations. 

**Figure 5:**
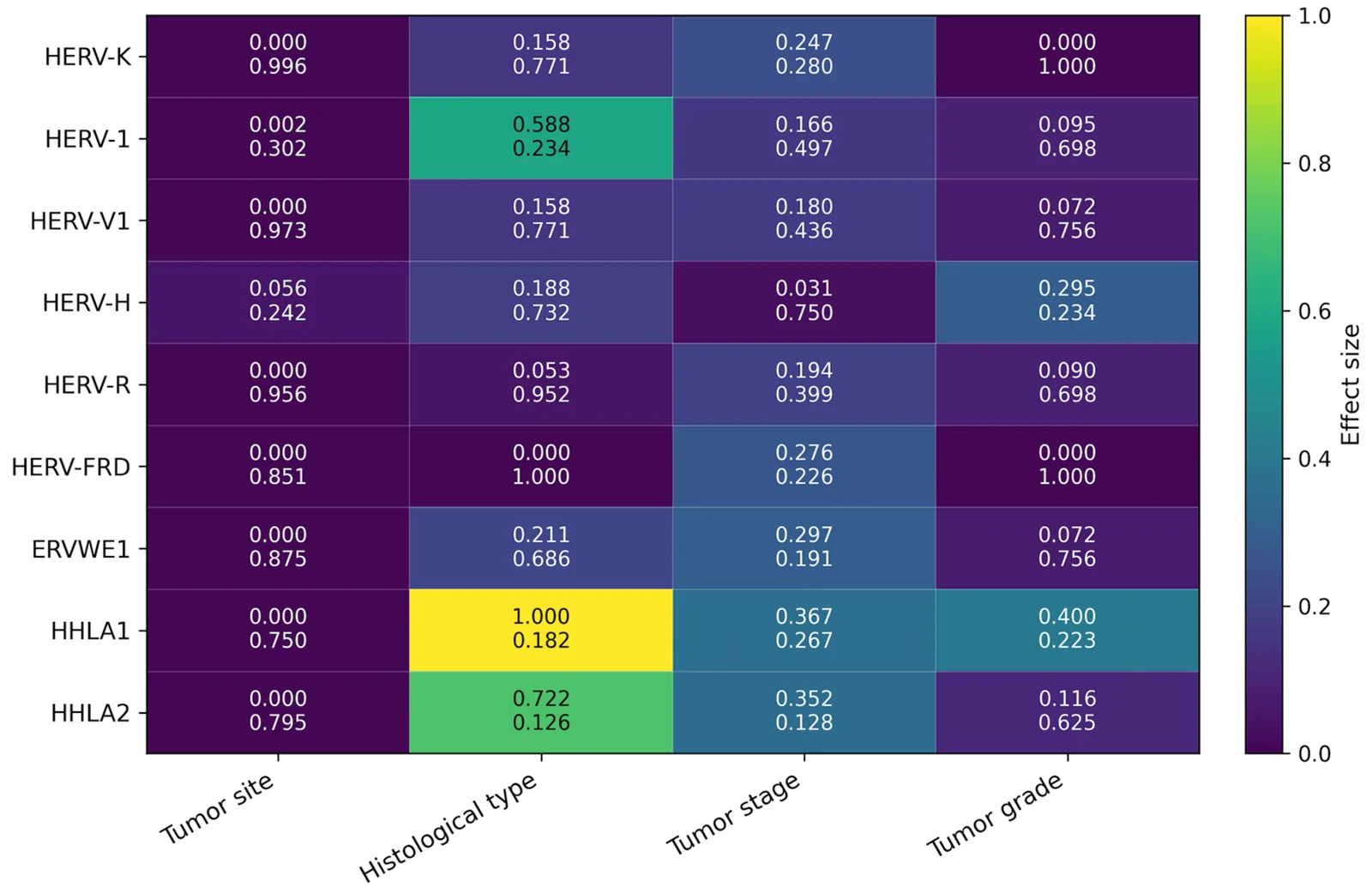
Associations between the expression levels of HERV-1, HERV-V1, HERV-K, HERV-H, HERV-FRD, HERV-R, ERVWE1, HHLA1 and HHLA2 and clinicopathological variables (tumor site, histological type, tumor stage (I–IV), and tumor grade (G1–G3)). Color intensity reflects the magnitude of the effect size (ε^2^ for comparisons involving more than two groups—tumor site; |rank-biserial r| for two-group comparisons—histological type; |Spearman ρ| for ordinal variables—stage and grade). The corresponding *p* value is indicated within each cell.

Subsequently, we explored associations between tumor tissue expression of the investigated HERV-associated transcripts (HERV-K, HERV-1, HERV-V1, HERV-H, HERV-R, HERV-FRD, ERVWE1, HHLA1, and HHLA2) and clinicopathological variables (tumor site, histological type, tumor stage, and tumor grade) (Figure 5). Overall, no robust associations were identified between tumor expression and the assessed clinicopathological characteristics, with most comparisons showing small effect sizes and non-significant *p*-values. Tumor site did not reveal meaningful expression differences for any transcript, with effect sizes that were essentially negligible (Figure 5). Likewise, for tumor stage and tumor grade, associations were generally weak to moderate and not statistically significant across the marker set. A single comparison showing a relatively larger effect size was observed for HHLA1 in relation to histological type (|r| ≈ 1.0); however, this association did not reach statistical significance (Figure 5), suggesting that the signal may reflect imbalanced subgroup distributions and/or a limited number of eligible observations. Taken together, these results indicate that, within this exploratory cohort, variation in tumor expression of the analyzed transcripts does not consistently align with the evaluated clinicopathological phenotype.

## 4. Discussion

The paired comparative analysis of tumor tissue and matched tumor-free adjacent tissue revealed a distinct pattern of HERV expression deregulation in CRC. Among the investigated families, HERV-FRD, HERV-R, HERV-K, HERV-V1, ERVWE1, HHLA1 and HHLA2 demonstrated increased expression in tumor tissue, whereas HERV-1 and HERV-H did not exhibit consistent differences between the two tissue types. These findings support the hypothesis that HERV reactivation in CRC does not represent a uniform global phenomenon but rather appears to involve a selective subset of HERV-associated transcripts, potentially reflecting locus-specific epigenetic regulatory mechanisms within the neoplastic context. This interpretation is consistent with previous reports describing heterogeneous HERV expression profiles across different tumor types [22,23]. HERV expression profiles differ substantially according to tumor type and the specific retroviral family investigated. HERV-K expression has been reported in a broad spectrum of malignancies, including breast cancer, melanoma, germ-cell tumors, leukemia, colorectal cancer, Hodgkin lymphoma, lung cancer, and soft-tissue sarcoma [22]. Other HERV families show more restricted or distinct tumor-associated patterns; for example, HERV-H has been described in liver, lung, and testicular tumors, while HERV-W/ERVWE1-related expression has been reported in several solid malignancies [22]. Moreover, comparative analyses of HERV Env proteins demonstrated heterogeneous expression of HERV-K and HERV-R across different tumor types, with differences in both the frequency and intensity of expression [23]. These observations indicate that HERV reactivation is not a uniform pan-cancer phenomenon but depends on tumor type, the specific HERV family and locus, and the underlying epigenetic and transcriptional context.”

An additional argument supporting the selective nature of HERV reactivation lies in the predominantly locus-specific behavior of these retroelements, which is influenced by the activity of long terminal repeat (LTR) promoters and chromatin accessibility at particular genomic insertions [24]. Furthermore, accumulating evidence indicates that HERV expression is closely linked to epigenetic processes, including DNA methylation and histone modifications [25,26].

The observed overexpression of HERV-FRD suggests that this family may constitute a component of the tumor transcriptional landscape. Diaz-Carballo et al. reported both increased HERV-FRD expression in colorectal tumors and its association with chemoresistance [27]. Similarly, the convergent expression patterns observed for HERV-R, HERV-K, and HERV-V1 further support the possibility of coordinated activation of specific HERV-associated transcripts in CRC. These retroelements have previously been implicated in colorectal carcinogenesis [28,29,30,31]. For instance, Ko et al. proposed that the HERV-K env gene may influence cellular proliferation, migration, and tumor colonization via a nuclear protein-1 (NUPR1)-dependent pathway [28]. Other studies have described increased expression of HERV-K env, gag, and pol transcripts in CRC, highlighting its potential relevance as an immunotherapeutic target [31]. At the tumor cell level, active HERV expression may contribute to the stimulation of innate immune responses, thereby influencing tumor immunogenicity and potentially modulating responsiveness to immunotherapeutic strategies [31,32]. Golkaram et al. demonstrated that HERV expression, in conjunction with CD8+ tumor-infiltrating lymphocyte density, may have prognostic value, including associations with recurrence risk and chemoresistance [24].

ERVWE1, which encodes the envelope protein syncytin-1, is frequently reactivated in CRC as a consequence of epigenetic dysregulation [33,34]. Its expression has been associated with tumor cell fusion, formation of polyploid giant cancer cells, and potential metastatic progression [35]. Experimental studies have suggested that ERVWE1 overexpression may promote cell cycle progression, proliferation, migration, invasion, and alterations in genes involved in epithelial–mesenchymal transition [34,35]. Additionally, syncytin-1 has been implicated in chemoresistance through activation of the MEK/ERK signaling pathway [36].

Data regarding HHLA2 in CRC remain controversial. For example, Kula et al. recently reported HHLA2 overexpression in colorectal tumor tissue in 97% of cases [37]. The authors further described a positive correlation between the percentage of HHLA2 expression and the tumor-infiltrating lymphocyte (TIL) score, along with a negative correlation between HHLA2 expression, anti-tumor cytokines, and pro-tumor growth factors [37]. Based on these findings, they proposed a potential role for HHLA2 as an immune checkpoint in CRC [37]. In this model, HHLA2 expression within tumor tissue may contribute to T-cell inactivation through binding to the KIR3DL3 receptor, thereby facilitating immune escape [37]. In contrast, Zhang et al. reported low HHLA2 expression in CRC [38]. Notably, in our dataset, despite the upregulation of HHLA2 and HHLA1 in tumor tissue compared with matched tumor-free adjacent tissue, HERV-H showed only a modest positive tumor–normal difference that did not reach statistical significance. One possible explanation is that HHLA1/HHLA2 transcripts may be more stable within the tumor microenvironment, whereas the polymorphic HERV-H–derived RNA may be more prone to rapid degradation. Additionally, this finding may suggest the concept of onco-exaptation, whereby tumor cells repurpose specific HERV-H–derived sequences to gain a selective advantage [39]. Further studies are required to clarify these hypotheses.

Subsequent paired analyses of IL-17 cytokines revealed increased tumor expression of IL-17E. Although other IL-17 family members exhibited descriptively higher expression levels in tumor tissue, these differences did not reach statistical significance. IL-17D was not detected in any analyzed sample. This observation may be interpreted in light of studies suggesting that IL-17D interacts with CD93 rather than canonical IL-17 receptors, influencing IL-22 production by mature group 3 innate lymphoid cells (ILC3) [40]. Reduced IL-17D signaling may impair IL-22-dependent antimicrobial peptide expression, potentially facilitating intestinal dysbiosis and contributing to a pro-inflammatory environment associated with tumorigenesis [40].

An observation of our study was the expression of IL-17A in conventional-type adenocarcinomas (NOS), predominantly in tumors with moderate differentiation and rectosigmoid localization, irrespective of tumor stage. The rectosigmoid segment harbors a particularly dense and distinct bacterial community, with enterotoxigenic *Bacteroides fragilis* being one of the bacteria frequently identified at this level [41]. This bacterium releases *Bacteroides fragilis* toxin (BFT), which induces a T-helper 17 (Th17) immune response, leading to increased IL-17A expression and recruitment of myeloid-derived suppressor cells (MDSCs), thereby contributing to colorectal carcinogenesis [42]. In our previous study assessing plasma IL-17A concentrations, this cytokine was detected at very low levels, suggesting that its potential clinical relevance in CRC may lie more in tissue-associated expression than in its circulating plasma levels [43].

However, given the limited number of cases in which IL-17A was detected, this observation should be interpreted cautiously and considered hypothesis-generating.

Regarding IL-17B, expression of this cytokine was identified predominantly in tumor tissue, regardless of tumor location. The predominance of IL-17B expression in tumor tissue is consistent with evidence suggesting that the IL-17B/IL-17RB signaling axis may support tumor cell survival, proliferation, and remodeling of the tumor microenvironment (TME), indicating a primarily local rather than diffuse mucosal role [44]. Additionally, IL-17F expression was detected exclusively in colorectal tumor tissue. This finding is consistent with the study by Chen et al., who reported significant overexpression of IL-17F in tumor mucosa compared with paired non-tumoral mucosa [45]. The same authors also demonstrated the involvement of IL-17F in promoting cellular migration and invasion through the induction of epithelial–mesenchymal transition (EMT) [45].

IL-17E (also known as IL-25) has been described as exerting context-dependent effects, with both antitumoral and protumoral roles depending on the immune microenvironment, tumor stage, and histological subtype [46]. In sporadic CRC, particularly in APC-mutant settings, increased IL-17E expression has been associated with protumoral effects and unfavorable prognostic features [34]. Proposed mechanisms include modulation of ILC2 responses, expansion of MDSCs, suppression of type 1 immunity, and promotion of angiogenesis, stromal remodeling, and tumor invasion [46,47,48].

In the second part of our study, correlation analyses identified statistically significant associations between the paired differential expression of selected HERV-associated transcripts (HERV-FRD, HERV-K, HERV-R, HERV-V1, and ERVWE1) and IL-17E, and these correlations remained significant after FDR correction. In contrast, HERV-associated transcripts did not show statistically significant correlations with NFκB expression. From a biological perspective, endogenous retroviral transcripts may promote IL-17E induction through activation of specific pattern-recognition receptors (PRRs), engaging upstream innate-sensing pathways that do not necessarily converge on canonical NFκB signaling, but may instead preferentially activate transcriptional programs mediated by factors such as interferon regulatory factors (IRFs) or STAT family members. Within this framework, IL-17E may contribute to the maintenance of a pro-tumoral microenvironment by facilitating myeloid-derived suppressor cell (MDSC) recruitment/expansion and suppressing T cell–mediated anti-tumor immunity [40,41]. Conversely, the lack of significant co-variation between HHLA1/HHLA2 and IL-17E suggests that these HERV-associated immune checkpoint–related transcripts may be governed by distinct regulatory drivers within the tumor context, whereas the selectively upregulated HERV/ERVWE1 subset appears to track with an IL-17E–linked inflammatory milieu. Collectively, these findings support the hypothesis that crosstalk between inflammatory signaling, epigenetic dysregulation, and endogenous retroviral reactivation may represent a biologically relevant axis in CRC [22,25,26]. Given the exploratory nature of this study, these findings should be interpreted as hypothesis-generating, suggesting a potential link between the differential expression of selected HERV transcripts and the IL-17E axis in CRC, which warrants validation in independent cohorts.

No statistically significant associations were observed between HERV expression and clinicopathological variables. These findings should be interpreted with caution, given the limited sample size and cohort composition.

Regarding the novelty of our study, it resides in the comparative evaluation of the expression profiles of multiple HERV- associated transcripts using paired tumor– tumor-free adjacent tissue samples, as well as in the integration of these findings with the expression patterns of IL-17 family cytokines and NFkB in patients with CRC. To the best of our knowledge, this is among the first paired-tissue studies in CRC to integrate HERV-associated transcript profiling with IL-17 family cytokines and NF-κB-related signaling. In contrast to most previous investigations, which have typically examined either HERV reactivation or inflammatory signaling in isolation, our data suggest a potential co-activation involving a subset of HERV- associated transcripts (HERV-FRD, HERV-K, HERV-R, HERV-V1 and ERVWE1) together with the IL-17E axis.

Our study has several limitations that should be considered when interpreting the findings. First, the relatively small sample size may limit the statistical power of the analyses, while the non-uniform distribution of cases—characterized by the predominance of stage II tumors and moderately differentiated (G2) lesions—may have contributed to the underestimation of potentially relevant biological associations. The limited cohort size also reduced our ability to fully characterize inter-patient molecular heterogeneity and to perform adequately powered clinicopathological subgroup analyses. Therefore, the findings should be regarded as exploratory and hypothesis-generating and require validation in larger, independent, preferably multicenter cohorts. An additional limitation is related to the intrinsic heterogeneity of bulk colorectal tissue samples. Both tumor tissue and matched adjacent non-tumorous tissue may contain variable proportions of malignant epithelial cells, non-malignant epithelial cells, immune infiltrates, stromal components, and endothelial cells. Therefore, the RT-qPCR results should be interpreted as global tissue-level transcriptional patterns rather than cell-specific expression signals.

In addition, the present investigation was based exclusively on transcriptomic assessments, without complementary functional or protein-level validation. Consequently, it cannot be established with certainty whether the observed gene expression alterations translate into measurable biological effects within the tumor microenvironment. Nevertheless, prior functional data support the biological relevance of HERV dysregulation in CRC [26]. In this regard, Ko et al. performed an HERV-K env knockout in DLD-1 colorectal cancer cells using the CRISPR-Cas9 system and demonstrated reduced cell proliferation, migration, invasion, and tumor colonization, together with decreased NUPR1 expression and lower ROS-related signaling, supporting an active role for HERV-K in CRC progression [26]. In addition, evidence from other disease settings supports the plausibility of a link between HERV activation and IL-17-related inflammation. For example, increased HERV-K expression in critically ill COVID-19 patients was associated with IL-17-related inflammation, monocyte activation, and unfavorable clinical outcomes, further suggesting that HERV-K upregulation may occur in parallel with pro-inflammatory immune programs. 

An additional methodological limitation is that agarose gel verification of the final qPCR amplicons for all analyzed targets was performed only for randomly selected samples. While the specificity of amplification was assessed by melting-curve analysis, randomly selected end-point amplicons agarose gel electrophoresis and the use of commercially pre-validated primers, additional confirmation of amplicon identity, such as sequencing, could enhance the validation of the results.

Importantly, the correlations identified between HERV-associated transcripts and IL-17E remain associative in nature, as mechanistic relationships were not explored. Accordingly, these correlations should not be interpreted as evidence of causality or direct pathway activation. Similarly, the absence of significant associations involving NFκB should not be considered definitive evidence of a lack of biological involvement, particularly in the context of the limited sample size and transcript-level assessment. An important limitation derives from the locus-specific nature of the RT–qPCR assays used to evaluate HERV-related transcription. The analyzed markers reflect the expression of selected HERV-associated loci/transcripts rather than global family-wide HERV activity. Consequently, the results should not be interpreted as exhaustive measurements of entire HERV families. An additional methodological limitation is the use of a single housekeeping gene (GAPDH) for normalization, without parallel validation against other reference genes in the present cohort. Although GAPDH is widely used in RT–qPCR studies, the inclusion of multiple validated reference genes would have provided a more robust normalization strategy. Finally, the cross-sectional design of the study precludes the evaluation of gene expression dynamics in relation to tumor progression or therapeutic interventions.

## 5. Conclusions

In this exploratory paired CRC cohort, tumor tissue showed selective upregulation of a HERV-associated transcript set (HERV-FRD, HERV-R, HERV-K, HERV-V1, ERVWE1, and HHLA1/2), whereas HERV-1 and HERV-H did not show consistent tumor–normal differences. IL-17E was also increased in tumors, and Δ(T−N) values demonstrated coordinated co-variation between IL-17E and the tumor-upregulated HERV/ERVWE1 axis after FDR correction, while NFκB did not co-vary robustly with this signature. Collectively, these findings support a candidate viral mimicry-related inflammatory hypothesis in CRC, in which selective HERV/ERVWE1 re-expression may contribute to a localized IL-17E-associated tumor microenvironment. Notably, although HHLA1 and HHLA2 were upregulated, their paired differential expression did not co-vary significantly with the main tumor-upregulated HERV/ERVWE1 signature or with IL-17E, suggesting partially distinct regulatory behavior within the tumor context. Although this interpretation remains indirect and requires mechanistic validation, it provides a more specific conceptual framework for future studies in this field.

Overall, our results suggest that endogenous retroviral elements and immune-related cytokines may participate in the complex molecular landscape of colorectal carcinogenesis, supporting further investigation of HERV-linked inflammatory signaling as a potential source of biomarkers and therapeutic targets in CRC. Given the limited cohort size, these findings should be regarded as exploratory and hypothesis-generating and require validation in larger, independent cohorts.

## Figures and Tables

**Table 1:** Forward and reverse primer sequences used for RT-qPCR analysis (https://www.origene.com).

Target	Primer Sequence (5′–3′)
HERV-FRD-F	ACCAGCTACCTGGGCATATCAG
HERV-FRD-R	GAGGCATTGGTGAATCGACTGG
ERVWE1-F	CGCAACTGCTATCACTCTGCCA
ERVWE1-R	CAGACAGTGACTCCAAGTCCTC
HERV-H -F	GTCTTGTCAACGGAAAGGGGTC
HERV-H R	CTTTGCTTGGGCTCAGAGGTCT
HERV-K-F	GCACTGATCCAACAGGAAAGGC
HERV-K-R	TCTGCTCCAGTGTCTACCAACC
HERV-R-F	CCTTTACAGACCCAGTAGGAGAG
HERV-R-R	AGAGAATGGGCTTGGGTGTGGT
HERV-V1-F	GGTGGAGTCTGTGCAGTGATCA
HERV-V1-R	GACTTCACAGCCTCCCAAATGG
HHLA1-F	ACAGAGGAGACCCTGAACACAG
HHLA1-R	GCTGTCAACCACTGAGTTCTGG
HHLA2_F	GGATTTTGGTGCCCTCTGCGAT
HHLA2-R	TCTTGTTGGGCTCCATCAGCAG
HERV1-F	GTGAGGAGTGTGCTGAAGACCT
HERV1-R	TGAGCAGTCGAAGTCAGGCTTG
IL17A-F	CGGACTGTGATGGTCAACCTGA
IL17A-R	GCACTTTGCCTCCCAGATCACA
IL17E-F	AACCGCCACCCAGAGTCCTGT
IL17E-R	ACAGGCAACGGGCGTGGTACA
IL17B-F	GCTGTGGATGTCCAACAAGAGG
IL17B-R	TCCTGCATGGTGAAGGGGTTCA
IL17F-F	AACCAGCGCGTTTCCATGTCAC
IL17F-R	GAGCATTGATGCAGCCCAAGTTC
IL17C-F	GCCCTCAGCTACGACCCAGTG
IL17C-R	AGCTTCTGTGGATAGCGGTCCT
IL17D-F	TGAGCAGGCGCGCAACGCGA
IL17D-R	GCAGTAGGCTTCAGGCAGGTAC
NFκB-F	GCAGCACTACTTCTTGACCACC
NFκB-R	TCTGCTCCTGAGCATTGACGTC

**Table 2:** Demographic, clinical, and paraclinical characteristics of the study cohort. Categorical variables are presented as *n* (%); age is presented as minimum, maximum, and mean.

Variable	Category	*n* (%)/Value
Sex	Female	12 (57.1%)
	Male	9 (42.9%)
Age (years)	Min	25
	Max	86
	Mean	64.2
Tumor location	Right colon	6 (28.6%)
	Left colon	9 (42.9%)
	Rectum	6 (28.6%)
Histological type	Conventional-type adenocarcinoma (NOS)	19 (90.5%)
	Adenocarcinoma (NOS) with mucinous component	2 (9.5%)
Tumor grade	G1	2 (9.5%)
	G2	17 (81.0%)
	G3	2 (9.5%)
Pathological TNM stage (pTNM)	I	4 (19.0%)
	II	9 (42.9%)
	III	4 (19.0%)
	IV	4 (19.0%)
Clinical characteristic	*n* (%)	
Smoking status	5 (23.8%)	
Alcohol consumption	8 (38.1%)	
Type 2 diabetes mellitus	8 (38.1%)	
Arterial hypertension	11 (52.4%)	
Dyslipidemia	4 (19%)	
Chronic coronary syndrome	2 (9.5%)	
Cardiac arrhythmias	2 (9.5%)	
Osteoporosis	2 (9.5%)	

**Table 3:** Comparative IL-17 and NFκB gene expression profiles between colorectal tumor tissue (T) and matched tumor-free adjacent tissue (N). Medians for T and N were calculated among quantifiable samples (≥LOD) only. Δ(T−N) represents the within-pair differential expression, and FC (median) was calculated as 2^Δ. McNemar exact (*p*) compares paired detectability (quantifiable vs. <LOD) between T and N. Wilcoxon paired (*p*) refers to the Wilcoxon signed-rank test for paired expression values, performed only in pairs with quantifiable values in both tissues. NA indicates that the corresponding statistic was not estimated due to an insufficient number of eligible pairs for that analysis. ND indicates values below the assay limit of detection (LOD). For analytes with few quantifiable values, medians should be interpreted cautiously.

n, 42 Samples (21 Paired Tumor Normal Tissues)	Median T Among Quantifiable Values (≥LOD)	Median N Among Quantifiable Values (≥LOD)	Median Δ(T−N)	FC (Median)	McNemar Exact (*p*)	Wilcoxon Paired *p*
IL-17A	8.953	8.239	0.714	1.643	0.031	NA
IL-17B	10.928	8.463	2.465	5.521	1.000	NA
IL-17C	5.883	3.808	2.075	4.213	0.500	0.352
IL-17D	ND	ND	NA	NA	NA	NA
IL-17E	4.392	1.923	1.102	2.147	1.000	0.001
IL-17F	11.138	ND	NA	NA	0.250	NA
NFκB	1.890	1.637	0.253	1.192	1.000	0.744

**Table 4:** Spearman correlations between paired differential expression values Δ(T−N) for HERV-associated transcripts and the investigated molecular/inflammatory biomarkers. Multiple-testing correction was performed using the Benjamini–Hochberg false discovery rate (FDR) method. FDR was applied across all correlations.

HERV (Δ T−N)	Marker (Δ T−N)	ρ (Spearman)	*p*	*p* (FDR)
HERV-K	HERV-FRD	0.968	<0.001	0.006
HERV-V1		0.953	<0.001	0.006
HERV-1		0.288	0.318	0.623
HERV-H		0.371	0.235	0.539
HERV-R		0.814	<0.001	0.006
ERVWE1		0.732	<0.001	0.006
HHLA1		−0.371	0.468	0.690
HHLA2		−0.217	0.359	0.650
IL-17E		0.825	<0.001	0.006
IL-17C		0.486	0.035	0.105
NFκB		0.229	0.413	0.665
HERV-V1	HERV-R	0.931	<0.001	0.006
HERV-1		0.121	0.681	0.836
HERV-H		0.336	0.286	0.588
HERV-K		0.945	<0.001	0.006
ERVWE1		0.742	<0.001	0.006
HHLA1		−0.257	0.623	0.818
HHLA2		−0.226	0.339	0.623
IL-17E		0.722	<0.001	0.006
IL-17C		0.253	0.115	0.281
NFκB		0.236	0.588	0.807
HERV-1	HERV-V1	0.380	0.180	0.483
HERV-H		0.517	0.085	0.230
HERV-K		0.953	<0.001	0.006
ERVWE1		0.735	<0.001	0.006
HHLA1		−0.257	0.623	0.818
HHLA2		−0.228	0.348	0.633
IL-17E		0.835	<0.001	0.006
IL-17C		0.550	0.018	0.066
NFκB		0.147	0.615	0.818
HERV-1	HERV-K	0.288	0.318	0.623
HERV-H		0.371	0.235	0.539
ERVWE1		0.732	<0.001	0.006
HHLA1		0.029	0.957	0.983
HHLA2		0.202	0.393	0.660
IL-17E		0.811	<0.001	0.006
IL-17C		0.488	0.034	0.105
NFκB		0.193	0.491	0.699
HERV-1	ERVWE1	0.099	0.737	0.870
HERV-H		0.406	0.191	0.498
HHLA1		0.433	0.056	0.162
HHLA2		0.034	0.833	0.948
IL-17E		0.560	0.013	0.043
IL-17C		0.181	0.216	0.525
NFκB		0.147	0.615	0.818
HERV-1	HHLA1	0.400	0.600	0.818
HERV-H		0.700	0.188	0.498
HHLA2		0.300	0.624	0.818
IL-17E		0.800	0.200	0.506
IL-17C		0.371	0.468	0.690
NFκB		0.020	0.950	0.980
HERV-1	HHLA2	0.099	0.749	0.873
HERV-H		0.073	0.832	0.948
IL-17E		0.091	0.710	0.856
IL-17C		0.014	0.956	0.983
NFκB		0.218	0.435	0.679
HERV-H	HERV-1	0.117	0.765	0.873
IL-17E		0.573	0.066	0.178
IL-17C		0.147	0.648	0.828
NFκB		0.321	0.365	0.650
IL-17E	HERV-H	0.261	0.467	0.690
IL-17C		0.455	0.159	0.441
NFκB		0.233	0.546	0.772
IL-17E	IL-17C	0.157	0.345	0.633
IL-17E	NFκB	0.214	0.443	0.679
IL-17C	NFκB	0.319	0.289	0.588

## Data Availability

The original contributions presented in this study are included in the article. Further inquiries can be directed to the corresponding author.

## Abbreviations

The following abbreviations are used in this manuscript:

APC	adenomatous polyposis coli
BFT	Bacteroides fragilis toxin
CRC	colorectal cancer
CRISPR–Cas9	clustered regularly interspaced short palindromic repeats–CRISPR-associated protein 9
DNA	deoxyribonucleic acid
EMT	epithelial–mesenchymal transition
ERVWE1	endogenous retrovirus group W member 1 envelope
FC	fold change
FDR	false discovery rate
GAPDH	glyceraldehyde-3-phosphate dehydrogenase
HERV	human endogenous retrovirus
HHLA1	HERV-H long terminal repeat-associating protein 1
HHLA2	HERV-H long terminal repeat-associating protein 2
IL	interleukin
ILC2	group 2 innate lymphoid cells
ILC3	group 3 innate lymphoid cells
IQR	interquartile range
IRF	interferon regulatory factor
LOD	limit of detection
LTR	long terminal repeat
MDSC	myeloid-derived suppressor cell
NA	not available/not applicable
ND	non-detectable
NFκB	nuclear factor kappa-light-chain-enhancer of activated B cells
NOS	not otherwise specified
NUPR1	nuclear protein 1
PRR	pattern-recognition receptor
RNA	ribonucleic acid
ROS	reactive oxygen species
RT–qPCR	reverse transcription quantitative polymerase chain reaction
SD	standard deviation
STAT	signal transducer and activator of transcription
TBK1	TANK-binding kinase 1
Th17	T-helper 17
TIL	tumor-infiltrating lymphocyte
TME	tumor microenvironment
TNF-α	tumor necrosis factor alpha
TNM	tumor–node–metastasis
UPR	unfolded protein response
WHO	World Health Organization
